# Robotic Anesthesia: How is it Going to Change Our Practice?

**DOI:** 10.5812/aapm.16468

**Published:** 2014-01-23

**Authors:** Arthur Atchabahian, Thomas M Hemmerling

**Affiliations:** 1 Department of Anesthesiology, NYU School of Medicine, New York, USA; 2 Department of Anesthesia, McGill University, Montreal, Canada

**Keywords:** Decision-Making, Computer-Assisted, Anesthesia, Robotics

In the relatively brief course of its history since the days of open ether inhalation, anesthesiology has undergone multiple radical or incremental changes. Endotracheal intubation and the introduction of muscle relaxants, continuous EKG monitoring, pulse oximetry and capnography, less toxic, shorter acting agents, processed EEG monitoring, and ultrasound guided regional anesthesia, among others, have completely transformed our practice. We would not conceive today of administering an anesthetic without access to these technological advances. Computerized recordkeeping is in the process of freeing practitioners from the rote task of copying to paper data that computers can easily store. Yet progress has been rather slow compared for example to computer science or aviation: only 65 years elapsed between the Wright brothers’ first flight and both a supersonic commercial airplane and man walking on the moon.

While most practitioners are aware of the progress of robotic surgery, especially for prostatic surgery, robotic anesthesia has gained rather little exposure until now. Impressive progress has been made, however, such as closed loop systems (1), intubating robots (2) or regional anesthesia robots (3). Despite uncertainty on how to measure all components of anesthesia, and especially analgesia (some researchers are using derivatives from the bispectral index, such as the variance of the BIS value or the EMG component, although it is unclear how these reflect clinically acceptable surrogates of pain (4)), closed loop systems will actually enter clinical practice very soon. The Sedasys system, that administers propofol sedation titrated to the processed EEG and vital signs to patients undergoing endoscopy without direct supervision by an anesthesia provider, was recently approved by the Food and Drugs Administration in the United States. As industrial robots, once relegated to working behind fences lest they injure humans standing in the wrong place, are fitted with sensors and safety systems that allow them to work alongside humans, we cannot help thinking that these “collaborative robots” will soon be assisting us in our daily tasks in the operating room.

The first question most colleagues ask when robotic anesthesia is discussed is “are we going to lose our jobs?” Most artificial intelligence specialists speculate on the occurrence of the Singularity, the time at which computers will match then surpass human intelligence, and predict it to occur sometime between 2030 and 2045. While the broad consequences of such an event are unpredictable and beyond the topic of this editorial, this would make human anesthesia providers redundant; however, that would be true of most other sectors of human activity. Ultimately, we might lose our jobs, but so will everyone else.

The current priority is to address the question of how those changes will impact our daily practice. Technological progress has constantly upset societal order. For example, Luddites in the 19th century destroyed the first mechanical looms that they thought threatened their livelihood. The Industrial Revolution transformed first England, then most of the Western world, beyond recognition. Closer to us, the rise of computers, the internet, mobile telephony and data connections has changed our daily life to an extent that was in the realm of science fiction only a few decades ago. The technological improvements in the field of anesthesiology, noted above, have made anesthesia significantly safer. However, we must also recognize that they have led to a loss of clinical skills among younger practitioners, who tend to rely on tests and monitors rather than examining the patient.

While robotic assistance for anesthesia is being rolled out, we can focus on those tasks that humans perform better than computers. Robots can help human practitioners improve care by increasing their precision and reliability, aiding their vigilance, and freeing them up to focus on higher level tasks and procedures. Humans are flexible and are better at problemsolving than machines, but they take poorly to repetitive tasks that quickly lead to boredom, fatigue and a drop in vigilance as well as low morale. The assumption of researchers is that robotic assistance during anesthesia will make our profession more enjoyable and even safer by decreasing the menial aspects that machines do well, simplifying the documentation, and allowing us to focus on the patient rather than the equipment and the paperwork. The risk, obviously, is overreliance on the technology and a paradoxical drop in vigilance. Ergonomics, i.e. adjusting the environment to the needs of the humanrobot team, might help reduce that risk by providing feedback in a form that is informative yet not overwhelming, and highlights the essential.

Economic considerations might include a reduction in the cost of care, provided that the cost of the equipment decreases enough due to economies of scale, and a need for fewer “higher level” practitioners (physicians rather than nurse anesthetists or anesthesia assistants) per patient. However, as the population ages and more surgical procedures are performed, that should not involve a decrease in the number of positions available.

Experience with industrial robots shows that while workers initially fear losing their jobs, companies often end up hiring more personnel because production costs drop. Workers warm up quickly to the robots and, as they do the programming themselves, tend to see the robots as subordinates rather than a threat. Research is also ongoing on the ways to improve robot acceptability and likability. For anthropomorphic robots, gestures accompanying speech increase their likability. Interestingly, mildly incongruent gestures, suggesting that the robot could make mistakes, made the robot even more likable (5). Whether that is desirable in a medical setting is debatable.

The question is “How can robotic anesthesia enter the daily practice, in which useful and structured way, allowing a smooth transformation from the present state of development towards the future of anesthesia?” One could envision a 3 step introduction:

## 1. Development and Introduction of Decision Support Systems

Currently, decision support systems are hardly available in anesthesia, despite many studies showing that these systems can help us to do a better job, and limit human mistakes (6). Some of these systems have been tested in various situations where limited vigilance leads to a constant level of insufficient performance, from missed alarm settings to missed drug administration, the most striking being the administration of preemptive antibiotic prophylaxis. Focusing on ‘smart alarms’, these decision support systems could easily be implemented in current perioperative monitoring systems or anesthesia information management systems. Let us take an example: how often do we forget to monitor neuromuscular blockade, either during surgery or at the end? An honest answer would be quite often. Why is there no ‘smart monitoring system’ that would ask us at the end of surgery to monitor neuromuscular blockade, suggest the correct site (the adductor pollicis muscle), maybe even show it on the screen, ask us to enter the value and then suggest or recommend a line of action, according to current guidelines? Why are such systems not available when every clinician would agree that they add value to anesthetic safety in order to avoid postoperative residual paralysis?

We need to develop and integrate these systems to make anesthesia even safer.

## 2. Automated Assist Devices: Semi-Automated Anesthesia

Closed loop administration of propofol is well established; independent from the parameter used to measure depth of anesthesia, more than two decades of research have shown that these systems can deliver propofol automatically, efficiently and safely. One could easily envision having an infusion pump able to deliver propofol guided by an anesthesia depth monitor. A similar system could be applied to the administration of volatile anesthetics; so far, no such system has been tested despite the technical requirements being available in anesthesia machines such as the Zeus (Drager) anesthesia machine (7). These monitoring parameters, the bispectral index being the most commonly used, reliably reflect depth of anesthesia and can be used and are used in daily routine to guide the administration of anesthetic drugs, such as propofol or volatile anesthetics.

## 3. Completely Automated Anesthesia

The introduction of automated anesthesia systems in clinical practice is not limited by their performance or their safety but only by the present regulatory hurdles. In the light of the recent FDA approval of the rather controversial Sedasys system, a semi-automated propofol delivery system that will be used by non-anesthetic health care providers (gastroenterologists) controversial because, at this stage, a machine is unable to adjust the level of sedation to the anticipated discomfort of discrete parts of the procedure, or to the specific skill and speed of a given operator, but also because, if a patient gets overly sedated, as is possible even with an experienced practitioner, Sedasys will not lift the jaw or ventilate the patient using a face mask, and there is no reversal agent for propofol one might question the validity of NOT making automated anesthesia delivery systems, tested in thousands of patients, available for use by anesthesiologists, who are experts in anesthesia delivery. Regulatory agencies need to reassess their attitude towards robotic or automated anesthesia systems.

For better or for worse, we cannot resist technological advances. Our role is to manage those advances to best benefit patients, but also to avoid disappearing, like travel agents and bank tellers, who were displaced by the Internet. Taking ownership of the technology is paramount: we need to be the drivers of progress rather than those who resist it out of inertia.
